# Molecular Diagnostics for Invasive Fungal Diseases: Current and Future Approaches

**DOI:** 10.3390/jof10070447

**Published:** 2024-06-26

**Authors:** David Pham, Varsha Sivalingam, Helen M. Tang, James M. Montgomery, Sharon C.-A. Chen, Catriona L. Halliday

**Affiliations:** 1 Centre for Infectious Diseases and Microbiology Laboratory Services, Institute of Clinical Pathology and Medical Research, NSW Health Pathology, Westmead Hospital, Westmead, NSW 2145, Australia; david.pham@health.nsw.gov.au (D.P.);; 2 Faculty of Medicine and Health, The University of Sydney, Camperdown, NSW 2006, Australia; 3 Sydney Infectious Diseases Institute, The University of Sydney, Westmead, NSW 2145, Australia

**Keywords:** culture-free diagnostics, whole genome sequencing, fungal metagenomics, antifungal resistance, fungal PCR, molecular diagnostics

## Abstract

Invasive fungal diseases (IFDs) comprise a growing healthcare burden, especially given the expanding population of immunocompromised hosts. Early diagnosis of IFDs is required to optimise therapy with antifungals, especially in the setting of rising rates of antifungal resistance. Molecular techniques including nucleic acid amplification tests and whole genome sequencing have potential to offer utility in overcoming limitations with traditional phenotypic testing. However, standardisation of methodology and interpretations of these assays is an ongoing undertaking. The utility of targeted *Aspergillus* detection has been well-defined, with progress in investigations into the role of targeted assays for *Candida*, *Pneumocystis*, *Cryptococcus*, the Mucorales and endemic mycoses. Likewise, whilst broad-range polymerase chain reaction assays have been in use for some time, pathology stewardship and optimising diagnostic yield is a continuing exercise. As costs decrease, there is also now increased access and experience with whole genome sequencing, including metagenomic sequencing, which offers unparalleled resolution especially in the investigations of potential outbreaks. However, their role in routine diagnostic use remains uncommon and standardisation of techniques and workflow are required for wider implementation.

## 1. Introduction

Invasive fungal diseases (IFDs) represent a significant, growing global health threat [1] with expansion of the at-risk population [2]. Notably, there is an increasing proportion of immunocompromised patients, with novel and more intensive immunosuppressive agents being introduced [3]. Furthermore, new risk factors for IFDs are evident, such as poorly controlled diabetes mellitus or co-infections with pathogens such as SARS-CoV-2 or tuberculosis. Increasing antifungal drug resistance, as well as limited therapeutic options, represent treatment challenges [4], with attributable mortality from IFDs estimated at 40–90% [5]. From a One Health lens, climate change has also driven the evolving epidemiology of IFDs [6]. Early and accurate diagnosis is essential to inform appropriate targeted antifungal therapy.

Given the limitations with traditional histopathological and culture-based diagnostic approaches, molecular methods play an increasingly important role for IFD diagnosis [7,8,9,10]. Nucleic acid amplification tests (NAATs) such as PCR assays have been used for decades to offer increased sensitivity and decreased turnaround times (TATs). However, standardisation of both pathogen-specific and broad-range PCR and the inclusion of these techniques into diagnostic algorithms has been addressed only more recently [11,12].

The increasing accessibility of next-generation sequencing (NGS) or whole-genome sequencing (WGS) techniques has also led to studies into fungal genomics and offers unparallelled resolution in antifungal resistance, intra-host evolution and epidemiological investigations into outbreaks, all of which are well described in the literature [10]. The use of metagenomic NGS (mNGS) from direct patient specimens has also afforded diagnosis of IFDs which would have otherwise been missed [13]. Nevertheless, there remain significant barriers to the use of NGS and mNGS techniques in diagnostic laboratories. 

In this review, we present a contemporary update of molecular techniques in fungal diagnostics and review emerging concepts in pathology stewardship, assay standardisation and future directions for research. We also briefly discuss the fundamentals of fungal DNA extraction protocols which underpin the success of any molecular-based assay. 

## 2. Fungal Nucleic Acid Extraction

High-quality and sufficient yield of fungal nucleic acid is paramount for any fungal NAAT. There is significant heterogeneity in yield depending on the specimen type, disease syndrome and extraction method used. It is important to minimise nucleic acid degradation, such as by preferentially testing fresh tissue rather than formalin-fixed paraffin-embedded (FFPE) specimens (see Section 3). Testing of specimens where fungal elements are visualised by microscopy also increases diagnostic yield by NAAT [14]. Other considerations include whether to extract cell-free or organism-sourced nucleic acid, or both [15]. For further detail, we refer readers to a comprehensive review by White et al. [10].

One notable goal of standardisation has been to harmonise the extraction processes used for pathogen-specific assays, which historically have accounted for significant variations between laboratories [16]. Different methods, such as physical, chemical or enzymatic extraction methods can produce highly variable yields of fungal nucleic acid depending on specimen type and pathogen [17]. These methods include, but are not limited to, non-enzymatic or enzymatic lysis, mechanical bead beating, spin column extraction, or heat-based extraction, to self-contained extraction systems such as the BioFire^®^ FilmArray^®^ [18]. For mNGS purposes, due to the relative abundances of host and pathogen nucleic acid within clinical specimens, approaches to depleting host nucleic acids or enriching pathogen nucleic acids (or both) should be considered [19]. Due to the significant heterogeneity amongst sample types and clinical syndromes, to date most standardisation efforts have focused on *Aspergillus* (discussed further in Section 4.1) [16].

## 3. Broad-Range Molecular Assays

Broad-range or panfungal PCR assays use universal fungal primers to amplify part of the multi-copy rRNA gene cluster (usually the internal transcribed spacers 1 and 2 (ITS1 and ITS2) or the D1/D2 regions of the 28*S* rRNA gene) followed by DNA sequencing or high-resolution melt (HRM) curve analysis for identification of amplified targets [7,20,21,22,23]. These assays are increasingly being utilised for definitive identification of fungal pathogens from a wide variety of clinical specimens including blood, fresh tissue and FFPE tissue. Other than increased sensitivity and specificity compared with conventional culture-based methods, they can detect novel and unexpected pathogens that may not present with an obvious clinical syndrome [8,22,24]. 

The diagnostic yield has varied widely across studies [25,26]. An analysis of 823 clinical specimens by in-house panfungal PCR in a retrospective study undertaken by Kerkhoff et al. found differing yield based on sample type. The authors correlated the 58 PCR-positive results with patient and other microbiologic data, to assess the influence of these tests on patient management. The diagnostic utility of PCR-positive results was superior in tissue specimens when compared to those obtained from liquid or fluid media with particularly low yield in cerebrospinal fluid (CSF). Of note, *A. fumigatus* was the most common fungal species detected [27,28]. Another study comparing the utility of panfungal PCR with culture on sinus tissue from predominantly immunocompromised hosts reported sensitivities of 85.0% (95% CI, 70.1 to 94.3%) and 67.5% (95% CI, 50.9 to 81.4%, *p* = 0.1136), respectively. PCR was able to detect fungi not cultured in 14.9% of cases (8 of 54) and informed clinical decision-making in 16.7% of cases [29]. Although TATs were faster, culture remained a major contributor to clinical management. This study is congruent with the initial validation of a panfungal PCR by Lau et al. in which the assay performed well on specimens where fungal elements were visualized but no pathogen was grown [30].

Non-invasive sampling typically results in lower yield by panfungal PCR compared to sterile-site specimens. A single-centre paediatric study reviewed the utility of such a PCR over 10 years on 286 samples. Most amplified sequences from bronchoalveolar lavage fluid (BALF) samples (16/18) were deemed not clinically significant, whereas the two clinically significant results were from lung biopsy tissue, where *A. fumigatus* and *Acremonium* spp. were detected [20]. These findings are in keeping with those of Garnham et al. in which BALF samples sent with no clinical suspicion of IFD often yielded mixed fungal species or non-pathogenic fungi such as *Candida* spp. [25]. Camp et al. demonstrated similar findings, where their panfungal assay on respiratory samples exhibited a 39.6% discordance rate. Their series also demonstrated the limitation of panfungal on even deep airway samples as 21.9% of samples had partial concordance due to mixed culture results [23]. An additional retrospective review of the clinical utility and cost effectiveness of broad-range PCR tests including panfungal PCR for a range of sterile and non-sterile sample types over a 10-year period found 40/294 tests analysed were PCR-positive, but only nine influenced patient management. This was calculated as 3227 USD per positive test and 14,341 USD per change in patient management, highlighting the need for laboratory stewardship to limit testing to high yield sites [21]. 

When performed on FFPE tissue with features of fungal rhinosinusitis, panfungal PCR has a sensitivity of up to 87.5% with a corresponding specificity, positive predictive value (PPV) and negative predictive value (NPV) of 89.2%, 92.7% and 85.2% respectively [31]. A cost analysis study in Australia reviewed 20 months of FFPE panfungal requests. Of these, 248 samples were processed with only 45 showing fungal elements, of which 22/45 were positive, with only 16 (35.6%) being deemed clinically significant [26]. Of 203 samples showing no fungal elements, 19/203 were PCR-positive and only 6 (3.0%) were clinically significant. Average cost per clinically significant result was 258.13 AUD in the histopathology-positive group and 3105.22 AUD in the histopathology-negative group.

As an alternative, PCR-based assays alongside HRM analysis offer potential for rapid and cost-effective fungal identification without the requirement for post-amplification sequencing [8]. Rather than multiplexed probes and primers, the use of DNA intercalating dyes with sequence-specific melting temperature allows for species-level discrimination and also has the advantage of detection of mutations associated with antifungal resistance [32]. The most common HRM assays for IFD diagnosis are the FDA-approved Biofire^®^ FilmArray^®^ Meningitis/Encephalitis ME Panel and BCID Panel (both bioMérieux, Marcy-l’Étoile, France), which can identify *Cryptococcus neoformans*/*gattii* (both ME and BCID panels), *Candida albicans*, *Nakaseomcyes glabratus* (formerly *C. glabrata*), *Pichia krudriavzevii* (formerly *C. krusei*), *C. parapsilosis*, *C. tropicalis* and *Candidozyma auris* (formerly *C. auris*) (BCID panel only). Evaluations have demonstrated good overall agreement with culture [33,34]. However, neither assay detects all fungal genera and are expensive (129 USD per pouch in 2016 [35]). 

Whilst there is increasing use of broad-range assays, standardisation of methods and their inclusion into diagnostic definitions remain ongoing areas of work. The International Society of Human and Animal Mycology (ISHAM) Fungal PCR Initiative (FPCRI; www.fpcri.eu) working group initially set out to standardise *Aspergillus* PCR testing [11], but has since broadened its scope to include standardisation of DNA extraction and PCR detection of *Candida*, *Pneumocystis* [36], the Mucorales [37] and fungi in FFPE tissue. Importantly, the European Organisation for Research and Treatment of Cancer-Mycoses Study Group Education and Research Consortium (EORTC/MSGERC) Consensus Definitions of IFD, have included broad-range PCRs as part of diagnostic criteria for IFDs [12]. They recommended that broad-range PCRs should only be performed on FFPE tissue where fungal elements are present [26,38], by laboratories with sufficient experience [39,40]. Sequences should be compared against curated databases such as the Westerdijk Fungal Biodiversity Institute (https://wi.knaw.nl/Pairwise_alignment, accessed on 26 June 2024) or MycoBank databases (https://www.mycobank.org/Pairwise_alignment, accessed on 26 June 2024), which include the ISHAM database [41]. The molecular identification of the fungus should be consistent with histopathological morphology.

## 4. Pathogen-Specific Molecular Assays

Pathogen-specific molecular assays for the diagnosis of IFDs are often targeted to the most common pathogens, or those of greatest clinical significance. The utility of these assays can range from screening of early infection to diagnosis, and they are typically performed directly on patient specimens, offering faster TATs compared with traditional microscopy and culture [8]. However, it is important to note that molecular assays remain complementary to traditional techniques and are still required, especially when pathogen-specific PCR assays yield a negative result. *Aspergillus* PCRs have seen the greatest clinical uptake and have had the most rigorous standardisation by the FPCRI, resulting in its subsequent inclusion into EORTC/MSGERC guidelines, while assays for other fungi have seen varying degrees of utilisation, implementation and standardisation [12,42].

### 4.1. Aspergillus

Invasive aspergillosis (IA) is the condition where fungal molecular assays have been most well established, both in screening of early infection as well as diagnosis, and on respiratory as well as blood specimens [12]. Considerable efforts by the FPCRI have helped to standardise methodology, quality control and clinical validation [42]. Challenges included standardising specimen types, extraction methods and PCR targets, as well as its performance for screening and diagnosis, in comparison and addition to other techniques for the diagnosis of IA, such as galactomannan antigen. Commercial *Aspergillus* PCR assays are now well established in clinical care (Table 1). The combination of commercial *Aspergillus* PCR assays with the FPCRI methodology provides a fully standardised procedure which can be replicated outside mycology reference centres [42]. Many assays detect *A. fumigatus* only as this is the most common cause of IA; however, FPCRI recommends use of pan-*Aspergillus* assays, with the caveat that this may raise the potential for cross-detection of other genera. The targeting of a multicopy gene enhances the analytical sensitivity, with the 18*S*/28*S* rRNA and ITS regions being frequently targeted [43].

Methodological standardisation, principally around optimal nucleic acid extraction techniques, was a key consideration in the incorporation of *Aspergillus* PCR in the EORTC/MSGERC consensus definitions of IA [42]. There is robust evidence for performing *Aspergillus* PCR on serum, plasma and whole blood for IA diagnosis [12]. Sample volume (≥3 mL blood, ≥0.5 mL serum/plasma) and nucleic acid elution volume (<100 µL) were critical for whole blood, serum and plasma sample types. Testing serum or plasma for circulating DNA was methodologically straightforward compared with whole blood, as automated extraction platforms could be used [15,44,45]. Whole blood and plasma samples tended to offer the highest sensitivity, although serum samples were the most specific. 

*Aspergillus* PCR testing on blood can be used for both screening as well as diagnosis. The most recent Cochrane review from 2019 evaluated 29 primary studies corresponding to 34 data sets from 2000–2018 and concluded that PCR has a moderate diagnostic accuracy when used for screening IA in high-risk patients but note that the high sensitivity confers a high NPV [45]. Screening strategies are best applied in patients at high risk of developing of IA, such as transplant patients, neutropaenic patients, or patients admitted to the intensive care unit with influenza or COVID-19 [42,46]. Within non-neutropaenic patients, the limited angioinvasion of IA limits the utility of high-frequency screening. 

Cycle threshold (Ct) values from real-time quantitative PCR provide utility when interpreting the significance of a positive result, with blood samples typically producing late positives [15,47]. Interpretation of late Ct values remains difficult. Cruciani et al. [48] report in a systematic review that the use of mould-active antifungal therapy reduces the specificity of *Aspergillus* PCR, but does not affect the sensitivity, postulating that this may be a consequence of prophylaxis limiting the clinical progression of IA.

*Aspergillus* PCR testing on respiratory samples other than BALF has insufficient evidence to be included as part of IA diagnostic criteria [12]. Compared to extraction from blood, serum or plasma, the FPCRI has yet to finalise nucleic acid extraction recommendations for testing of BALF [42]. For instance, due to sample viscosity, some specimens may need liquefaction prior to extraction to allow for manipulation. Mechanical lysis of a BALF pellet may be required to extract organism-sourced DNA, while adding BALF supernatant will allow for free DNA to be targeted within the same nucleic extraction procedure [15]. Due to the invasiveness of the procedure, BALF samples are not used as screening for IA. 

Huygens et al. report [49] no difference in six week mortality for patients with isolated PCR positivity on BALF compared to patients with no mycological evidence of IA, noting that patients with isolated PCR results tended to have higher Ct values. However, there were insufficient patients with an isolated PCR result and Ct values below a 33.11 cut-off to provide a meaningful comparison of this subpopulation. An umbrella review by Cruciani et al. covering eight systematic reviews from 2007 to 2023 evaluating the performance of *Aspergillus* PCR for the diagnosed of IA in immunocompromised patients noted for BALF specimens a mean sensitivity ranging from 0.57 to 0.84 and mean specificity ranging from 0.92 to 0.97 with very low to low certainty due to heterogeneity and risk of bias in the primary studies [11]. The use of mould-active antifungal prophylaxis was noted to reduce the sensitivity of PCR in BALF, limiting the manifestations required to classify probable IA using EORTC/MSGERC definitions. 

Lamberink et al. have noted that the inclusion of *Aspergillus* PCR on BALF as a mycological criterion for probable IA in the EORTC/MSGERC definitions has led to an increase in the incidence of probable IA diagnoses. There were no differences in mortality between the probable and possible IA cases, suggesting that the increase in probable cases may reflect colonisation rather than infection. However, mortality was increased in cases with a low Ct compared to a higher Ct, using a cut off of 36.8 on their in-house assay. The sensitivity and specificity for 12-week mortality using this cut off was 75.0% and 61.7%, respectively [50]. 

Key future directions for research and development of *Aspergillus* PCR include broadening *Aspergillus* spp. detection beyond *A. fumigatus*, incorporating detection of molecular markers of antifungal resistance (discussed further in Section 5.2), as well as its use as a prognostic marker during therapy.

### 4.2. Pneumocystis jirovecii

Diagnosis of *Pneumocystis jirovecii* pneumonia (PCP) relies on visualisation of *P. jirovecii* by tinctorial or fluorescent staining and microscopy or detection of DNA by quantitative real-time PCR on a respiratory tract specimen or detection of β-D-glucan (BDG) in serum in the context of compatible host factors and clinical features [12]. Quantitative real-time PCR (qPCR) is preferred to qualitative PCR, which has been proposed to distinguish probable disease as opposed to colonisation. However, thresholds for positivity are not well defined [51]. Ct values have been suggested to distinguish colonisation from infection, although there is significant overlap between populations attributable to non-standardised collection techniques, variability between assays, variation in host factors and a wide dispersion of Ct values within a diagnostic category [36]. For instance, a comparison between the RealStar^®^ *Pneumocystis jirovecii* kit and an in-house assay found a high degree of concordance but noted 13.4% positive PCR results in the category of no final diagnosis of PCP [52].

The majority of commercial PCRs currently available target the mitochondrial large subunit (mtLSU) rRNA gene; the Roche LightMix modular *Pneumocystis jirovecii* PCR targets the major surface glycoprotein gene (MSG) and the PneumoGenius by PathoNostics B.V. targets both mtLSU and two specific dihydropteroate synthetase (DHPS) mutations which may be associated with sulfonamide treatment failure (Table 2) [53]. Other targets which have been reported include beta-tubulin, mitchochondrial small subunit (mtSSU), *Kex-1*, 5*S* rRNA, mitochondrial ribosomal rRNA and ITS [36]. 

The FPCRI recently conducted an international evaluation of five in-house and five commercial PCP PCR assays amongst 16 reference mycology laboratories to standardise PCP diagnostics [36]. Assays targeting both RNA and DNA—i.e., whole nucleic acid (WNA)—rather than DNA provided lower Cts, which was concordant with a similar cross-platform evaluation by Dellière et al. [54]. The mtSSU gene target provided lower thresholds than assays which targeted the mtLSU, MSG or beta-tubulin genes. Given the difficulties in choosing Ct values to define probable PCP versus colonisation, the working group suggested standardisation of a qPCR targeting the mtSSU gene as the basis for quantification of *P. jirovecii* nucleic acid burden. Target standardisation will offer more comparable Ct values across assays, which will improve inter-laboratory studies into interpretation of late Ct values representing either infection or colonisation. 

Other issues related to *P. jirovecii* testing include specimen types. Typically, BALF has been the specimen of choice; although the EORTC/MSGERC guidelines do not discriminate between different respiratory specimens, sensitivity of qPCR on upper respiratory tract samples is lower than on BALF [12,55]. Detection of *P. jirovecii* DNA remains highly specific for disease in meta-analysis, even for non-invasive sampling such as sputum or nasopharyngeal aspirates [51,56]. Induced sputum and BALF sensitivity and specificity exceeded 90%, whilst testing on nasopharyngeal aspirate, oral wash and serum specimens were less sensitive, but remained specific. Incorporation of BDG serum testing may assist in clarifying low-burden detections of PCP, whilst further research into appropriate qPCR thresholds in non-HIV-positive patients is required [55]. 

### 4.3. Cryptococcus

Mycological evidence for proven invasive cryptococcal disease includes culture of *Cryptococcus* spp. from sterile material, detection of cryptococcal antigen in blood or CSF, or detection of *Cryptococcus* spp. DNA from sterile specimens [12]. Species-level identification of *C. neoformans* complex and *C. gattii* complex is increasingly important due to their different clinical presentations, management and outcomes. 

Given the high sensitivity and specificity of cryptococcal antigen testing, as well as its accessibility, PCR-based diagnosis of cryptococcal disease has not been widely developed. Commercial assays for *Cryptococcus* are limited, although several in-house assays have been published (Table 3). Commercial assays have been hampered by inadequate sensitivity and may not discriminate between *C. neoformans* and *C. gattii* [57,58,59]. In these cases, culture and cryptococcal antigen testing appear more sensitive. In-house assays, on the other hand, can demonstrate high sensitivity and specificity as well as distinguishing between species by melt-curve analysis [60]. Of note, Mbangiwa et al. developed a species-specific qPCR to detect, identify and quantify *Cryptococcus* infections in patients with cryptococcal meningitis in sub-Saharan Africa. Compared to culture (*n* = 110), the sensitivity of pan-*Cryptococcus* 28*S* rRNA PCR on CSF pellets was 98.2%, while the sensitivity of the species-specific *QSP1* PCR assay was 90.4% at day 0, with quantification correlating to culture. The implications of persisting PCR positivity without persisting culture are uncertain [61]. Further research into the role of *Cryptococcus* PCR in comparison to cryptococccal antigen is required.

### 4.4. Candida and Candida-like Organisms

Molecular methods including PCR assays have demonstrated superiority in diagnosis of invasive candidiasis, significantly reducing time to diagnosis and directed therapy compared with blood culture for diagnosis of candidemia [62]. Whole blood is preferred over serum samples for diagnosis of candidemia with high sensitivity (95%) and specificity (92%) [63]; however, PCR positivity in blood may also be associated with other forms of invasive candidiasis. However, the sensitivity of PCR on whole blood for non-candidaemic deep-seated candidiasis is decreased compared to candidaemic patients [64,65]. This is likely due to the transient presence of candidemia associated with various forms of invasive candidiasis. Sensitivity of PCR on serum samples is lower (25%) when compared with BDG (94%) but is superior in specificity (91% compared with 29%) [66]. Combining multiple fungal biomarkers (e.g., PCR assays with BDG) is currently being explored for diagnosis of invasive candidiasis, including in the A-STOP trial (ISRCTN43895480). Clinical validation through the efforts of the FPCRI are underway to determine the optimal sample type (serum, plasma or whole blood) and further standardization of *Candida* PCRs (Table 4) [62].

Of commercially available *Candida* PCRs, T2Candida^®^ is the only FDA-approved assay with extensive clinical validation and has been included in the EORTC/MSGERC diagnostic option for probable invasive candidiasis [12]. The assay targets the ITS2 region with subsequent probe hybridization and magnetic resonance to detect five major pathogens currently or formerly classified under the umbrella of *Candida* spp. (*C. albicans*, *C. tropicalis*, *N. glabratus* (formerly *C. glabrata*), *Pichia kudriavzevii* (formerly *C. krusei*), *Candida parapsilosis*) [67]. Its advantages include a rapid TAT of 4–5 h, limited manual processing and low limit of detection (LoD) of 1–3 CFU/mL [68,69]. 

T2Candida^®^ is only validated for whole blood samples, and sensitivity may be affected by prior or concurrent use of antifungal therapy or absence of candidemia [8,70]. Other limitations include the high cost of upwards of USD 150 per test and the inability to distinguish between *C. albicans* and *C. tropicalis* or between *N. glabratus* and *P. kudriavzevii*, as well as its inability to detect other *Candida* spp. and traditional techniques are still important to avoid missing these pathogens [62,71]. In an early validation study looking at performance of T2Candida^®^ in detection of candidemia, an overall sensitivity of 91% and specificity of 99% were demonstrated on whole blood, but the majority of study samples were manually supplemented with clinically relevant titres of *Candida* spp. [69]. A recent systematic review and meta-analysis of T2Candida^®^ clinical performance revealed pooled sensitivity of 91% and specificity of 94% [70]. 

Separate to T2Candida^®^, T2Cauris™ panel detects *C. auris* with limit of detection of <5 CFU/mL for whole blood and skin surveillance swabs, with a TAT of 5 h, allowing for timely infection control measures [9]. Whilst costs can vary between countries, economic considerations have limited its uptake, especially compared to phenotypic screening with chromogenic media which can cost under 5 AUD per sample (*Candida* Plus, CHROMagar, Saint-Denis, France).

The Bruker Fungiplex^®^ *Candida* real-time PCR is another assay that has three different targets—*Candida* spp. (*C. albicans*, *C. parapsilosis*, *C. dubliniensis*, *C. tropicalis*), *N. glabratus* and *P. kudriavzevii*. Its performance for candidemia diagnosis was compared with blood culture and the Roche LightCycler^®^ SeptiFast real-time PCR in a study conducted on high-risk patients in intensive care units. The Bruker Fungiplex^®^ *Candida* assay demonstrated a sensitivity of 100% and specificity of 94% (*n* = 58), while the Roche LightCycler^®^ SeptiFast demonstrated lower sensitivity (60%) with comparable specificity (96.1%) [72]. There are limited validation data for other commercial *Candida* PCR assays.

### 4.5. Mucorales

Early diagnosis of Mucorales infections is challenging, due to limitations of conventional methods of histopathology and culture and the limited utility of serological assays. Recent advances in molecular techniques including pan-Mucorales and species-specific PCR assays offer promise in expediting diagnosis. Conventional and real-time Mucorales PCR assays targeting diverse biological samples such as serum, BALF and fresh and FFPE tissues have been described (Table 5) [73,74,75,76,77,78]. Real-time PCR assays are preferred due to their faster TAT, reduced contamination risk and potential to quantify fungal burden. These assays have predominantly targeted the ITS, 18*S* and 28*S* rDNA regions. *CotH*, a gene family of spore-coating encoding proteins, has recently been identified as an emerging diagnostic target as they are multicopy genes that are universally present in Mucorales and are specific for these fungi. Study of *CotH* presence in biological samples of infected mice illustrated better detection rates in urine over plasma and BALF. With an overall sensitivity of 90% and specificity of 100% (*n* = 126), *CotH* holds promise as a reliable biomarker for screening and detection of mucormycosis, although further validation in human cohorts is warranted [77]. Other targets undergoing further research include mitochondrial *rnl* gene and cytochrome b gene [79]. 

The FPCRI is prioritizing the standardization of Mucorales PCR. A recent interlaboratory evaluation demonstrated good performance and reproducibility of four different Mucorales PCR protocols [37]. A commercial pan-Mucorales PCR assay, the MucorGenius^®^ (PathoNostics, Netherlands), is a single-tube reaction that detects five Mucorales genus-specific targets (*Rhizopus* spp., *Mucor* spp., *Lichtheimia* spp., *Cunninghamella* spp., *Rhizomucor* spp.) and thus requires less reagent and manual processing. Upon its evaluation using serum from patients with culture-positive invasive mucormycosis in the prospective MODIMUCOR trial, PCR provided an earlier diagnosis by an average of eight days compared with conventional methods [80]. However, MucorGenius^®^ may miss low-burden infections, and its lack of species-specific targets is a disadvantage for diagnosis [79]. MycoGenie^®^ Real-time PCR kits (Adamtech, Pessac, France) detects both *Aspergillus* DNA and Mucorales DNA by targeting the 28*S* rDNA region. Prospective clinical evaluations have demonstrated 100% sensitivity using sera from patients with disseminated mucormycosis, including four patients with coinfections with *Aspergillus* spp. [81]. Further clinical validation of available assays may assist with standardization of Mucorales PCR.

### 4.6. Endemic Mycoses

The recent rise in incidence of endemic mycoses may be related to environmental changes, return of travel, increase in susceptible populations and improvements in diagnostics [22]. Histopathology requires expertise in interpretation, and culture-based methods require special precautions when handling culture isolates and demonstration of dimorphism for diagnosis. Furthermore, sensitivity of culture is low and often results in delay in diagnosis by several weeks due to the slow-growing nature of endemic fungi. Antibody testing may be unreliable in the immunosuppressed population, and while antigen testing can provide a rapid diagnosis, it is limited by poor specificity and cross-reactivity with other fungi [82]. Many endemic mycoses are listed on the WHO fungal priority pathogen list to guide research, development and public health action [1]. *Histoplasma* spp. is recognised as a pathogen of high priority, whereas *Coccidioides* spp., *Talaromyces marneffei* and *Paracoccidioides* spp. are listed within the medium-priority group. The European Confederation of Medical Mycology (ECMM) has an active working group aimed at performing multicentre studies on molecular diagnostic tools for endemic mycosis.

Molecular techniques such as loop-mediated isothermal amplification (LAMP), conventional PCR and qPCR can expedite diagnosis. Broad-range or panfungal PCRs are commonly used in non-endemic areas where there is a lack of targeted PCR or when there is no clear suspicion of a specific endemic mycoses causing the disease [22]. Specific PCRs for endemic fungi are primarily developed in-house by reference laboratories [22,83]. A multicentre external quality assessment of diagnostic assays for *H. capsulatum* and *Coccidioides* spp. revealed that targeted real-time PCR assays were more sensitive compared with broad-range PCRs and overall specificity was high (91.1% for *H. capsulatum* and 97.5% for *Coccidioides* spp.) [83]. 

Specific *Histoplasma capsulatum* PCRs have either targeted rDNA multicopy regions (e.g., 18*S* [84], ITS1/2 [85,86,87,88], mtSSU [89] or unicopy genes (e.g., 100-kDa-like protein or M antigen [90,91,92,93,94], *PPK* and *CFP4* [95]). A wide range of specimen types have been tested including respiratory secretions, biopsies, bone marrow, whole blood and serum. Sensitivity is often higher on specimens sampled at the site of infection. However, less invasive specimens may be preferred for diagnosis of disseminated infection [22]. Real-time PCRs have demonstrated superiority over conventional PCRs in terms of sensitivity and reproducibility [96]. Limited validation data on targeted *Histoplasma* PCRs have demonstrated variable sensitivity (67% to 100%) but high specificity (96–100%) [97]. 

Targets for *Coccidioides*-specific PCRs include ITS regions [98,99], Antigen 2 and Proline Rich Antigen [100]. Variable sensitivities (74–100%) have been reported on respiratory, fresh and FFPE tissue and CSF samples [101]. The GeneSTAT.MDx *Coccidioides* (DxNA LLC, St. George, UT, USA) was one FDA-approved PCR, with 100% sensitivity and 93.8–100% specificity; however, it is no longer marketed [102].

Most *Paracoccidioides*-specific PCRs are conventional PCRs, with only two real-time assays described. Many PCRs target the ITS region [103] or genes encoding proteins *Gp43* [104] or *Pb27* [105]. Respiratory samples, biopsies, blood and sera have been tested with sensitivities of 91–100% reported. 

Few assays have been described for detection of *Blastomyces* spp., targeting *BAD1* gene [106,107] or *DRK1*. High sensitivity and specificity have been reported in limited clinical studies. LAMP, conventional nested PCRs and real-time PCRs for *Talaromyces* spp. have been described targeting 5.8*S* [108], 18*S* [109], or ITS rDNA regions [110,111,112]. Overall sensitivity of 84% and specificity of 99% have been reported in range of specimens including plasma, blood, serum or bone marrow. Further clinical validation is warranted to reach consensus on extraction method, sample preparation and preferred PCR targets.

## 5. Molecular Detection of Antifungal Resistance

Antifungal susceptibility testing by phenotypic methods has utility for guiding antifungal treatment and for surveillance for drug resistance [113]. However, it is impacted by slow TATs and lack of sporulation for certain mould species, thus preventing in vitro testing and the absence of interpretive criteria for the majority of species. Resistance testing by molecular methods offers a rapid alternative to assist with clinical decision making, but also has limitations. The focus areas are in the detection of azole and echinocandin resistance of *Candida* and *Aspergillus* spp., but the techniques described are common to detection of resistance genes for any fungus-drug combination.

### 5.1. Candida and Candida-like Organisms

As azole or echinocandin drugs are the backbone of treatment of serious *Candida* infections, resistance-conferring mutations for these antifungals are more well-characterised. For azoles, the *ERG11* gene encodes the target enzyme, lanosterol 14 α-demethylase. Resistance to azoles is multifactorial and not only involves specific *ERG11* point mutations, but also upregulation of genes involved in drug efflux such as the ATP-binding cassette transporters and major facilitator superfamily transporter genes such as *Candida* Drug Resistance (*CDR1*) and Multi-Drug Resistance (*MDR1*) [114]. Mutations in the former alter the structure of lanosterol 14 α-demethylase thereby preventing azole binding whilst the latter result in reduced intracellular azole concentrations. On balance, *ERG11* and other *ERG* gene mutations appear to play a relatively minor role in azole resistance in most *Candida* spp., although there are notable exceptions, including the *ERG11* Y132F and R398I mutations in *C. parapsilosis* [115,116] and the *ERG11* mutations Y132F, K143R and V125A in *C. auris* [117]. 

Conversely, upregulation of efflux pumps is the result of the upregulation of *CDR1/CDR2* and *MDR1* genes through point mutations in the transcription factor genes *TAC1* and *MRR1*, which encode for efflux pumps. These are particularly important in *N. glabratus* strains that are resistant to azoles [118,119]. Mutations in *TAC1* gene are a major driver of azole resistance in *C. parapsilosis* [115]. 

All the above mechanisms of resistance have been described across various *Candida* spp. including *C. albicans*, *N. glabratus*, *P. kudriavzevii*, and *C. parapsilosis* as well as *C. tropicalis* and *C. auris* (Table 6).

Due to the diverse co-existing resistance mechanisms, in vitro susceptibility testing is a more accurate methodology for clinical laboratories in discerning azole resistance than genotypic testing [7,120,121,122,123,124]. This phenomenon was demonstrated in a recent study, in which *Candida* spp. isolated from vulvovaginitis samples with reduced susceptibility to azoles underwent amplification and sequencing of regions within *ERG11*, *TAC1*, *UPC2*, *MRR1* and *MRR2* genes in which resistance associated mutations had been described. The analysis revealed several previously well-described mutations but also novel mutations within *TAC1* and *MRR1* genes, underscoring the challenges in designing commercial assays for antifungal resistance detection [125]. 

Resistance to echinocandins is characterized by specific amino acid alterations in the FKS subunits, resulting in a thousand-fold reduction in enzyme sensitivity to the drug class. Most *Candida* spp. possess three *FKS* genes (*FKS1*, *FKS2* and *FKS3*), with mutations in *FKS1* primarily driving resistance, except in *N. glabratus*, where mutations in both *FKS1* and *FKS2* contribute to resistance [120]. The most common *FKS1* mutations occur in two highly conserved ‘hotspot’ (HS) regions: HS1 amino acid position 641–649 and HS2 position 1357–1364 with mutations in F641, S645 and R1361 having the most pronounced minimum inhibitory concentration (MIC) increase [126,127]. Identification of *FKS* mutations has been shown to correlate with clinical failure [128,129]. 

Hence, detection of genes known to confer antifungal resistance or novel resistance mutations is reliant on DNA sequencing. Whilst Sanger sequencing may suffice where a limited number of genes are being analysed, WGS provides a genome-wide view of gene mutations and is also able to detect new combinations of mutations that might otherwise be missed with targeted DNA sequencing [130,131,132]. Even for echinocandin resistance where clinical utility is best understood, no commercial methods are currently available. This limitation is mainly attributed to the need for sequencing after PCR amplification of *FKS* regions in current methods. Furthermore, as mutations can occur outside the hotspot regions, routine sequencing for diagnostic purposes may necessitate sequencing the entire *FKS* gene [133,134,135,136]. Similarly, for *ERG11* and transcription factor mutations, WGS is preferred. mRNA experiments to document gene overexpression are also required but are beyond the scope of the present review [137,138].

### 5.2. Aspergillus

Most studies of drug resistance in *Aspergillus* relate to azole resistance in *A. fumigatus* sensu stricto. The primary mechanism of azole resistance in *A. fumigatus* is caused by mutations in the *Aspergillus cyp51A* gene, analogous to the *ERG11* gene in yeasts. This results in decreased azole affinity for 14α-demethylase. Other mechanisms, such as induction of CYP51B expression, ABC family transporters and MFS transporters have been described, but their clinical relevance remains uncertain. The clear correlation between specific mutations in *cyp51A* and azole resistance in clinical isolates makes them excellent targets for PCR-based diagnostic tests [120]. The most commonly described azole resistance mutation worldwide is the 34 base tandem repeat in the promoter of the *cyp51A* gene and a leucine-to-histidine change at codon 98 (TR34/L98H); a 46 base insertion in the promoter region and amino acid change at codons 121 and 298 (TR46/Y121F/T289A) are also well described [139]. Non-synonymous point mutations can occur independent of tandem repeats at codons G54, M220 and G138 and G484 as detailed in Table 7. There remains a significant proportion of clinical *A. fumigatus* isolates demonstrating phenotypic triazole resistance but with a wild-type *cyp51A* gene. In one study out of Denmark, non-*cyp51A*-mediated resistance accounted for 19.7% (13/66) of all resistance [140]. This highlights the ongoing need for sequencing to further understand additional resistance associated mutations [141]. Echinocandin resistance in *A. fumigatus* is less well described and is detailed in Table 7 and requires further genomics surveillance to understand its role in resistance.

WGS has been used to monitor genomic variants in azole resistant *A. fumigatus* complex species worldwide in both clinical and environmental isolates. It is especially important for ongoing surveillance of non-*A. fumigatus* complex species as well as further delineating the role of membrane transporter protein gene and non-*cyp51A* mutations such as cholesterol import and HapE mutations [144,145,146]. Targeted sequencing with pre-amplification of tandem repeat regions can be performed, but the utility of these methods is unclear when non-*cyp51A* mediated triazole-resistant clinical isolates ranges from 15 to 60% [146,147].

There are currently three commercial assays which can detect the most common *cyp51A* mutations; however, sequence-based approaches are still used in the research setting (Table 8) [133,148]. It is important to recognise that the absence of a mutation on a commercial assay does not infer susceptibility due to the limited range of mutations detected [149]. A prospective study across 12 centres in the Netherlands tested the AsperGenius^®^ species and resistance PCR assay (PathoNostics, Maastricht, the Netherlands) [49]. Inclusion criteria were haematology patients with imaging findings of pulmonary IA with BALF sampling. Of 323 patients, there were only eight cases of probable IA where resistance-conferring mutations were detected. Six of the eight had a positive culture, but only four had phenotypic susceptibility testing performed. Resistance was confirmed in three out of the four samples tested. In the discrepant sample, despite the BALF culture demonstrating phenotypic azole susceptibility, a sputum sample 14 days later demonstrated phenotypic resistance [49]. A comparative performance of three different PCR assays in detecting *Aspergillus* DNA from BALF while evaluating the presence of *cyp51A* gene mutations for BALF samples of immunocompromised patients was conducted by Scharmann et al. of the 103 samples tested, only one showed phenotypic resistance, which was detected by all three assays [150].

With the increasing availability of molecular diagnostics, many cases of invasive aspergillosis are diagnosed in the absence of culture by nucleic acid amplification. These assays are often multicopy and more sensitive than the single copy *cyp51A* targets. Therefore, inability to amplify resistance genes in up to 30% of patients with azole resistant infection may occur. Lack of implication of a resistance gene does not imply susceptibility as this could be related to a mutation not tested or lack of sensitivity due to single copy number if used for direct detection from sample [133].

### 5.3. Other Fungi

The growing concern over terbinafine-resistant dermatophytes has prompted the creation of a PCR assay designed to detect mutations in the squalene epoxidase (*SQLE*) gene associated with missense mutations (Leu393Ser/Phe or Phe397Leu) [151]. Terbinafine inhibits fungal growth by blocking the activity of *SQLE*, resulting in the accumulation of squalene and the depletion of ergosterol from the fungal wall; mutations or overexpression of *SQLE* can result in terbinafine resistance. A recent study investigated the effectiveness of the commercial DermaGenius^®^ Resistance real-time PCR in identifying these mutations in isolates of *Trichophyton* spp. (Table 8). Sequencing of these isolates confirmed the presence of the specified mutations, and concordance was observed with the DermaGenius assay results. Furthermore, the mutations identified by both methods correlated with high minimum inhibitory concentration (MIC) to terbinafine, ranging from 16 to ≥32 mg/L, as determined by broth microdilution testing [152]. 

Trimethoprim/sulfamethoxazole remains the first-line treatment and prophylaxis for PCP, although most of the anti-*Pneumocystis* activity is thought to be conferred by sulfamethoxazole [153]. Mutations in dihydrofolate reductase (DHFR) can confer resistance to trimethoprim, while mutations in the dihydropteroate synthase (DHPS) gene can confer resistance to sulfamethoxazole [154]. Clinical evaluation of the PneumoGenius^®^ dihydropteroate synthase (DHPS) polymorphisms had full concordance with the in-house methodology used by Guegan et al. (Table 8). Mutations were found even in patients who had not had trimethoprim/sulfamethoxazole exposure and encompassed those with human immunodeficiency virus (HIV), haematological and solid organ malignancies, as well as prolonged steroid use [53].

**Table 8:** Commercially available molecular assays for antifungal resistance.

Fungal Pathogen	Antifungal	New Commercial Platforms
*Aspergillus* spp.	Triazoles	AsperGenius^®^ Resistance TR Multiplex real-time PCR [49,150] (PathoNostics, Maastricht, The Netherlands) *Aspergillus fumigatus* TR34 *Aspergillus fumigatus* TR46 *Aspergillus fumigatus* cyp51A (WT)—melt curve analysis allows detection of mixed infections AsperGenius^®^ G54/M220 RUO PCR detects G54 and M220 RUO in cyp51A of *A. fumigatus* [155] MycoGENIE^®^ *Aspergillus fumigatus* and TR34/L98H (Adamtech, Pessac, France) Fungiplex^®^ *Aspergillus* Azole-R IVD PCR (Bruker Daltonik GmbH, Bremen, Germany) *Aspergillus fumigatus* TR34 *Aspergillus fumigatus* TR46
Dermatophytes	Terbinafine	DermaGenius^®^ Resistance Multiplex real-time PCR [155] (PathoNostics, Maastricht, The Netherlands) *T. rubrum*/*soudanense* *T. interdigitale*/*mentagrophytes*, *T. mentagrophytes* (ITS type IV) *T. tonsurans* *T. violaceum* *Trichophyton quinckeanum*/*Trichophyton schoenleinii* SQLE alterations: Detected via melt curve analysis Leu393Phe, Phe397Leu (predominant mutations) Leu393Ser, Phe397Ile, Phe397Va
*P. jirovecii*	Trimethoprim/sulfamethoxazole	PneumoGenius^®^ [53] (PathoNostics, Maastricht, The Netherlands) Dihydropteroate synthase (DPHS) mutations (codon 55,57)

### 5.4. Overall Considerations

Molecular testing for antifungal drug resistance may be warranted in the following clinical scenarios [133]:

1. Clinical failure on appropriate antifungal therapy, experiencing a relapse or developing a new infection after prolonged antifungals.
2. High local resistance rates, e.g., *N. glabratus* to echinocandins or *A. fumigatus* to voriconazole.
3. Limited EUCAST/CLSI compliant phenotypic testing.
4. Borderline susceptibility/resistance results from phenotypic testing, where identifying resistance mechanisms can guide clinical decisions.

Validation of molecular methods are difficult to standardize. The genotype tested for needs to be linked to a validated mechanism of resistance to a drug which ideally also corresponds to a phenotypically detectable change that can be correlated. As the diversity of resistance phenotypes increases, including those not targeted by PCR, the correlation between PCR results and resistance phenotypes may become less defined [7,133,149].

## 6. Whole Genome Sequencing

WGS approaches in mycology have been slower to be established in routine clinical care compared to virology or bacteriology. In recent years, however, there has been increased interest in fungal genomics, due to advances in NGS technologies, decreased costs and overheads and improvements in analysis pipelines [156,157]. For diagnostic purposes, NGS can be performed from cultured isolates (discussed here) or from direct patient samples (discussed further in Section 7). Analysis of the fungal genome by NGS first and foremost offers increased resolution beyond conventional techniques for organism identification and characterisation. NGS also has important roles in epidemiological contexts including studies of fungal evolution, the mycobiome and host genomic susceptibility to IFDs, all of which are beyond the scope of this review. With respect to IFD outbreak investigations, we highlight some key developments, but also direct readers to recent comprehensive reviews [158,159]. 

Accurate identification of causative fungal pathogen is one fundamental area where NGS can provide benefits over conventional techniques, including morphology, biochemical tests or MALDI-ToF proteomics [160]. Gostinčar described the use of a simple and efficient pairwise genomic distance analysis using 16-mers in differentiating fungal species based on a minimally curated GenBank database, correctly distinguishing over 90% of analysed species [161]. Salem-Bango et al. outline the validation and implementation of an NGS-based fungal identification method based on the Illumina MiSeq platform, creating consensus ITS sequences which were then queried against the Westerdijk Fungal Biodiversity Institute database [162]. They demonstrated 100% (74/74) concordance of WGS identifications at the genus level and 89% (66/74) at species level. Of eight discordant identifications at species level, each was explained through limitations of conventional techniques or with taxonomic reclassifications. On the other hand, the TheiaEuk pipeline infers taxonomic assignments using a Genomic Approximation Method with a custom fungal database, similar to the GAMBIT method for bacterial identification [163,164]. The pipeline also has sub-workflows for *C. neoformans*, *C. auris* and *A. fumigatus*, performing antifungal resistance identification for all three species, as well as clade typing for *C. auris*. During validation, TheiaEuk correctly assigned genomes to species level for 126/135 (93.3%) and to genus level for 131/135 (97%) of instances. 

Yu et al. developed a targeted enrichment method for fungal identification on the Nanopore platform [165]. Over twenty-five thousand 120-mer probes were designed from Benchmarking Universal Single-Copy Orthologs (BUSCO) datasets and other fungal phylogenetics studies, evaluated in silico and then, as proof-of-concept, utilised to genomically characterise isolates of plant pathogens such as *Fusarium circinatum*. Accurate taxonomic assignment was based on majority rule consensus trees, with medium depth of coverage exceeding 6000×. However, further studies are required to evaluate its routine applicability to human IFD diagnosis.

NGS is also useful to characterise novel or emerging pathogens. The identification of *C. auris* was first confirmed and further characterised by NGS [166]. Initial attempts to identify this pathogen by phenotypic techniques had significant limitations with identifying *C. auris*, as it is closely related to other pathogenic *Candida* spp. such as *C. haemulonii* [167]. Santos et al. performed comparative genomic and phenotypic characterisation of emerging pathogens *A. lentulus* and *A. fumigatiaffinis* in comparison with *A. fumigatus*, identifying key species- and strain-specific polymorphisms in both *cyp51A* and *FKS1* and correlating these with phenotypic susceptibility and virulence testing [168]. Similarly, the emerging pathogen *Trichophyton indotineae*, often misidentified phenotypically as *T. mentagrophytes* or *T. interdigitale*, was recently genomically characterised, where the average nucleotide identity was calculated in comparison to other *Trichophyton* spp. and mutations were analysed in the squalene epoxidase (*Erg1p*) and the *cyp51A* and *cyp51B* genes (*ERG11* gene family), implicated in antifungal drug resistance [169]. 

Another key study area of NGS use is in the area of antifungal drug resistance. Whilst targeted assays looking at commonly described resistances genes have been developed (discussed further in Section 4), novel mechanisms of resistance often require further genomic characterisation [170,171]. Furthermore, NGS has been successfully used to track intra-host evolution of pathogens, especially in cases where antifungal resistance has developed in vivo [172]. Keighley et al. described breakthrough *C. albicans* bloodstream infection following in vivo development of a 14-bp deletion in the *ERG3* gene, resulting in pan-azole resistance. Genomic analysis of antifungal drug resistance remains complex, as novel mechanisms play a significant role, such as promoters introduced by transposable elements [173], or chromosomal monosomy [174].

The clinical utility of NGS within fungal outbreak situations is well established and comprehensively reviewed by Douglas et al. [158]. Its application in the case clusters of yeasts, *Pneumocystis*, mould and endemic mycoses have met with varying degrees of success, depending on the pathogen. Menu et al. describe an infection control response to a *Saprochaete clavata* (current name *Magnusiomyces clavatus*) outbreak in an oncology unit, where NGS was used to demonstrate genomic relatedness of clinical and environmental isolates, leading to identification of a deficient dishwasher heating system as the contamination source [175]. These isolates exhibited a ≤10 SNP difference. A *C. auris* outbreak in 2019 showed that 9/10 sequenced clinical isolates were related to an index case [176]. Di Pilato et al. utilised molecular clock analysis to predict a recent introduction, with the outbreak postulated to be related to a ward conversion to a COVID-19 ICU during the pandemic. The TheiaEuk pipeline was utilised alongside the Nullabor and MycoSNP pipelines to characterise the southern Nevada outbreak of *C. auris*, where it was able to uncover new introductions using shared SNP analysis [164,177]. Additionally, NGS also represents value in excluding point-source outbreaks. For instance, an ICU *A. fumigatus* outbreak was resolved by NGS genotyping, as a lack of genetic similarities suggested a construction-related source, rather than a shared clinical origin; the outbreak later resolved when construction ceased [178]. 

Despite the benefits of NGS in fungal diagnostics, however, there remains some key limitations to its widespread use. Whilst there are multiple methods of extracting nucleic acid from fungi, comparative studies of these methods are limited [179,180]. Importantly, extraction of high molecular weight DNA is essential in obtaining high-quality genomes via long-read sequencing [181]. Hybrid assemblies involving both long- and short-read NGS technologies are key to generating high quality reference genomes moving forward, although routine long-read sequencing availability is relatively limited compared to short-read technologies [182]. There are also a limited number of complete, high-quality reference genomes available for fungal pathogens [183]. Fungal genomes are larger, have more complex taxonomy and can exhibit polyploidy [184]. Misidentifications have been described in the literature [185], and our understanding of fungal taxonomy continues to evolve [186,187,188]. Initiatives such as MycoCosm [189,190], *Candida* Genome Database [191], Ensembl [192] and FungiDB [193] aim to increase the availability of fungal sequences and support comparative analysis. Likewise, bioinformatic analyses are often laborious and require significant experience. For instance, further experience is required for cluster definitions by SNP distance; while this is well defined for yeast outbreaks, delineating mould outbreaks is more difficult [158]. Finally, limiting factors associated computing power, storage space, expertise and cost are especially applicable for medical mycology applications [194]. As experience and cost overheads improve, however, NGS technologies will become increasingly available and applicable to the diagnosis of IFDs.

## 7. Fungal Metagenomics

Metagenomic next-generation sequencing or mNGS is rapidly moving from research to clinical laboratories. Similar to applications of NGS on cultured isolates, this has largely been focused on detection of bacteria and viruses, rather than fungi [195,196,197,198]. In principle, this catch-all approach has the capacity to detect all pathogens—bacteria, viruses, fungi and parasites—simultaneously in a clinical sample. The potential clinical applications of mNGS in medical mycology are the same as those for other infectious diseases diagnostics and include pathogen identification, antimicrobial resistance prediction, virulence characterization and microbiome analysis. Detailed descriptions of these applications and their relevant technologies are beyond the scope of this manuscript but are found in recent reviews [13,199,200]. In targeted mNGS approaches, one may select for detection of a particular pathogen group(s) for a given type of clinical specimen; however, drawbacks include bias towards detection of target microorganisms. More often, the clinical question is one of unresolved diagnosis with no pathogen identified by conventional methods and where the requirement to determine which organism is causing disease. Thus, untargeted or shotgun mNGS analyses may be preferred where the entirety of DNA and/or RNA is sequenced [201]. 

Early glimpses into the clinical utility of untargeted mNGS were promising. mNGS first provided clinically actionable information in the diagnosis of neuroleptospirosis in a 14-year critically ill boy with meningoencephalitis [198] and soon after became utilized in public health medicine to investigate outbreaks of foodborne illness and to survey antibacterial resistance in food supplies [202,203]. Its implementation into routine diagnostic mycology, however, has been more challenging; where a diagnosis has been made by mNGS, this was often in the context of syndromic testing for infection at a particular body site with the pathogen detected as a collateral finding. Most experience has been in the setting of respiratory infection; meningoencephalitis and sepsis though other body fluids have also been tested. There are case reports on using direct mNGS to diagnose specific fungal infections including *Talaromyces marneffei* [204]. 

### 7.1. Respiratory Tract Infection

As the airways are exposed to the atmosphere, the presence of filamentous fungi in non-sterile respiratory secretions can be difficult to interpret as to whether the fungus is a true pathogen, a coloniser or a contaminant. Defining specific microbial profiles that are diagnostic remains a challenge and findings need to be interpreted in clinical context.

The utility of mNGS on BALF to diagnose fungal pneumonia has been relatively well studied, with many reports stemming from China. In brief, mNGS has been reported to be more efficient than culture, pathogen-specific PCR and conventional staining [197,205,206,207] in diagnosing fungal lung infections. The yield may however depend on the population studied. Lin et al. studied 60 immunocompromised patients with a diagnosis of pneumonia; mNGS yielded a higher diagnostic accuracy of pulmonary fungal infection than conventional methods (78% vs. 57%), with *P. jirovecii* comprising the commonest pathogen (76.7%), followed by *Aspergillus* (14%) and *Cryptococcus* (9.3%) species [206]. In a similar study analysing 246 BALF, the sensitivity of mNGS was likewise significantly higher for fungal infection (78.5% vs. 39.3%) with good specificity. As above, the commonest pathogen was *P. jirovecii* (6.6% of cases) followed by *C. albicans* (6%) with nine patients with mixed bacterial–fungal infection [207]. Other than for isolation of *P. jirovecci*, the clinical relevance of isolation of other fungal species is not clear from the reports. Of note, Sun et al. found the sensitivity and specificity of BALF mNGS for PCP diagnosis in non-HIV immunosuppressed patients to be 97.4% and 85.1%, respectively [208].

Other respiratory specimens have been evaluated for diagnostic utility using mNGS. In one study of 59 community-acquired pneumonia (CAP) patients (104 samples), mNGS was undertaken using including BALF, pleural fluid, blood, CSF and urine. Compared with other diagnostic techniques, mNGS detected a greater number of pathogens and confirmed clinical management in 35/59 cases. Notably and again, more fungi were detected when mNGS was performed and *P. jirovecii* was the commonest pathogen (23.7%), although *A. fumigatus* and *C. albicans* were also identified with uncertain clinical significance [197]. Huang et al., in a study of largely immunocompromised patients with suspected pneumonia, retrospectively examined 467 BALF, brush biopsy specimens and lung biopsy specimens by mNGS in comparison with conventional diagnosis. Of 171 patients with a final diagnosis of pneumonia, 66 (38.6%) were deemed to have fungal pneumonia (*Aspergillus* *n* = 32, *P. jirovecii* *n* = 22, *Rhizopus* spp. *n* = 7) [205].

Other studies have examined the value of using lung biopsy samples for clinical mNGS in more depth. Of 133 patients with suspected pulmonary infections/abnormal imaging findings that underwent both short- and long-read mNGS analysis, the former showed 77.6% sensitivity and 97.6% specificity, respectively, compared to the reference diagnosis standard [209]. Long-read sequencing, however, showed 34.7% sensitivity and 98.7% specificity [209]. Notably, mNGS identified a greater number of fungi in lungs, confirmed by subsequent pathological examination, including four instances of *Aspergillus* and one *Saccharomyces* infection not detected by other methods. Wang et al. also used mNGS to study mixed pulmonary infections; mNGS identified a broader spectrum of pathogens with overall improved diagnostic sensitivity of pulmonary fungal infection; fungi (*Pneumocystis*, *Aspergillus* and *Rhizopus*) were detected in 31/55 vs. 10/55 by conventional methods [210]. 

A more recent study compared the diagnostic efficacy of histopathological testing alone with the combined use of mNGS and histopathology on core needle biopsy tissue for pulmonary infections. The combination approach detected more fungi (*Cryptococcus*, *Aspergillus* spp.) than histopathology alone and improved the detection rate for rare pathogens such as *Talaromcyes* spp. [211]. The areas under the receiver operating characteristic (ROC) curve of mNGS combined with histopathological examination for pulmonary lung infection was 0.876 compared with 0.76 for histopathology. 

### 7.2. Central Nervous System Infection

In a study of seven patients with subacute or chronic meningitis in which no diagnosis was made by conventional methods, four fungi, one each of *C. neoformans*, a fungus reported as *A. oryzae*, *H. capsulatum* and *C. dubliniensis* were identified in CSF as the plausible and likely cause of the illness [212]. Importantly, the testing of healthy patients as well as reagent control samples enabled the devising of an algorithm to effectively separate bona fide pathogen sequences from spurious environmental sequences. Whilst there are other diagnostic methods for cryptococcal meningitis and other causes of fungal meningitis are uncommon, clinical suspicion should be maintained in a patient with appropriate risk factors where standard diagnostic tests have not provided the diagnosis. 

### 7.3. Blood Stream Infection

Blood, as a common and convenient sample for laboratory testing, has also been studied by mNGS to detect bloodstream pathogens. Overall, several studies have demonstrated that early implementation of mNGS can effectively improve detection of pathogens in blood [196,213]. In one study, mNGS of plasma was compared with blood culture for 37 acute leukemia patients with febrile neutropenia. Of 14 causative bloodstream pathogens identified, nine were detected by mNGS only and five were detected by both mNGS and blood culture. However, only 1/37 patients had fungemia (*A. flavus*). Blood mNGS results of 1046 cases from a real-world cohort study found advantages of mNGS in detecting difficult to cultivate pathogens, although fungi were overall, uncommon. One notable finding was 55 cases of *P. jirovecii* were detected in blood of which 43 patients underwent targeted treatment [213].

### 7.4. Other Specimens

A recent French study reported on the prospective use of routine untargeted mNGS largely as a second-line testing approach on all sample types (*n* = 742) from 523 patients. A causative or possible causative pathogen was detected in 117 (25%) samples from patients with a high initial suspicion of infection versus 9 (3%) samples analysed to rule out infection, with good concordance (97%) with conventional tests. Only a very small proportion of pathogens were fungi, with no details provided [195]. Gu et al. developed a streamlined hybrid automated protocol for mNGS testing that was cross compatible with both long -read and short-read sequencing, suitable for all body fluids. The accuracy evaluation focused on the mNGS performance relative to conventional culture/PCR using cell free DNA to effect sequencing of 87 body fluids (abscess, joint, peritoneal, CSF, urine and BALF) from acutely ill patients. The number of fungi detected was small (*n* = 19; 18%) and the clinical significance of these reads was not stated [214]. Conversely a retrospective study of mNGS on various specimen types from 94 patients identified no advantage in fungal detection [215]. 

With regards to urine, in a single centre study of 33 patients from China, mNGS detected at least 1 pathogen in 29 (97.9%) cases, of which four instances of fungus (3 species—*Talaromcyes marneffei*, *C. parapsilosis* and *C. albicans* twice) were identified [216].

### 7.5. Challenges

The extensive data that can be leveraged from mNGS have many potential applications in medical mycology and may well enable a route to precision diagnostics. Systematic demonstration of its clinical utility in larger studies on varying specimen types and in different patient host groups is likely the largest major hurdle for routine clinical implementation. Nonetheless, there are several technical challenges that are worthy of mention. 

For untargeted mNGS, a problem can be the large amount of host DNA in clinical samples which can account for >99% of sequence reads, limiting overall analytical sensitivity. This can be mitigated by employing targeted sequencing, but with acknowledgment of the disadvantage of bias. More often, either depletion of human DNA, enrichment of pathogen DNA, or both, by application of adaptive sequencing are attempted. These processes are succinctly summarized by Hoang et al. [217].

Detection of microbial contaminants present in the sample, reagents or laboratory equipment can complicate analysis and result interpretation and strict quality control procedures are required. Well-characterised reference standards and controls are also needed to ensure quality and stability over time. Most available mNGS reference materials are customized to specific applications, such as the ZymoBioMICS Microbial Community Standard (Zymo Research, CA, USA) for bacterial and fungal metagenomics [218] and/or focused on a limited pathogen spectrum e.g., those that only contain bacteria and hence may not be appropriate for untargeted mNGS. Defining microbial profiles using standard protocols, that are predictive of disease can be difficult especially from non-sterile sites with a complex microbiome. Whilst several groups have successfully validated mNGS in Clinical Laboratory Improvement Amendments (CLIA)-certified clinical laboratories for the diagnosis of infections including meningitis/encephalitis, sepsis and pneumonia, none of the above focus on fungal pathogen detection [219,220,221].

Finally, user-friendly bioinformatics software for analysis of fungal mNGS data are not available. Thus, in house pipelines are typically required which necessitate highly trained personnel. Software packages such as SUPRI real-time [214], metaMaps, BugSeq and NGSpeciesID (summarised by Hoang et al. [217]) may be used but their fit with fungal identification is not defined. Design of a robust pathogen identification algorithm applicable for short, as well as long, fungal reads would be ideal together with a formula to calculate a normalized read per million [214]. These would inform criteria for reporting a positive result.

## 8. Conclusions

We have provided a contemporary update into molecular diagnostics of fungal infections, including broad-range and pathogen-specific molecular assays, as well as an exploration into the nascent utility of next-generation sequencing for medical mycology. With input from international consortiums such as EORTC/MSGERC and FPCRI, there has been increasing standardisation and uptake of these techniques for the diagnosis of IFDs. *Aspergillus* PCR has been well-investigated and incorporated into these diagnostic algorithms and NAATs for *Candida*, *P. jirovecii* and Mucorales are undergoing a similar evaluation and standardisation process. Standardisation for multiplex assays, broad-range PCRs and molecular diagnosis of antifungal resistance are sure to follow, but face increased challenges compared to single-target diagnostics. Furthermore, whilst NGS exhibits incredible potential for fungal diagnostics, current barriers include cost, turnaround time and expertise in interpretation. These represent critical avenues for future research and significant collaborative efforts are required to remain abreast of new developments and paradigm shifts.

## Figures and Tables

**Table 1:** Commercially available molecular assays for *Aspergillus* spp.

Assay	Manufacturer	Method	Target	Species	Samples
*A. fumigatus* Bio-Evolution	Bio-Evolution, Brysur-Marne, France	Real-time PCR	ITS1 region	*A. fumigatus*	Serum, BALF, sinus biopsy
artus^®^ *Aspergillus* diff. RG PCR	Qiagen, Düsseldorf, Germany	Multiplex real-time PCR	Target unknown	*A. fumigatus*, *A. terreus*, *A. flavus*	Blood
AsperGenius^®^ Species and AsperGenius^®^ Resistance	PathoNostics B.V, Maastrict, the Netherlands	Multiplex real-time PCR	28*S* rDNA	*A. fumigatus* complex, *A. terreus*, *Aspergillus* spp. TR_34_/L98H, Tr_46_/Y112F/T289A mutations	BALF, serum, plasma
*Aspergillus* spp. ELITe MGB^®^ Kit	ELITechGroup S.p.A, Turin, Italy	Quantitative real-time PCR	18*S* rDNA	*A. niger*, *A. nidulans*, *A. terreus*, *A. flavus*, *A. versicolor*, *A. glaucus*	BALF, aspirate, plasma
AspID	OlmDiagnostics, Newcastle, United Kingdom	Multiplex real-time PCR	Target unknown	*Aspergillus* spp., *A. terreus*	BALF, serum, plasma
FungiPlex^®^ *Aspergillus* and Fungiplex^®^ *Aspergillus* Azole_R	Bruker Daltonik GmbH, Bremen, Germany	Multiplex real-time PCR	Target unknown	*A. fumigatus*, *A. flavus*, *A. niger*, *A. terreus* TR_34_ and TR_46_ mutations	BALF, serum, plasma
LightCycler Septifast	Roche Diagnostics, Mannheim, Germany	Multiplex real-time PCR	ITS region	*A. fumigatus* (and *Candida* spp.)	Blood
Magicplex Sepsis Real-Time Test	Seegene, Seoul, Republic of Korea	Multiplex real-time PCR	Target unknown	*A. fumigatus* (and *Candida* spp.)	Blood
MycoReal *Aspergillus*	Ingenetix GmbH, Vienna, Austria	Real-time PCR with melt curve analysis	ITS2 region	*A. fumigatus*, *A. flavus*, *A. niudulans*, *A. niger*, *A. terreus*	BALF, blood, aspirate, CSF, tissue
MycoGENIE^®^ *Aspergillus* Species and MycoGENIE^®^ *Aspergillus fumigatus* and resistance TR_34_/L98H	Ademtech, Pessac, France	Duplex real-time PCR assay	28*S* rDNA	*Aspergillus* spp., *A. fumigatus* TR_34_/L98H mutations	Serum, biopsy, lower respiratory tract samples

BALF: bronchoalveolar lavage fluid; CSF: cerebrospinal fluid; ITS: internal transcribed spacer.

**Table 2:** Commercially available molecular assays for *Pneumocystis jirovecii*.

Assay	Manufacturer	Method	Target	Samples
RealStar^®^ *Pneumocystis jirovecii*	Altona Diagnostics GmbH, Hamburg, Germany	Real-time PCR	mtLSU	Unspecified
PneumoGenius^®^	PathoNostics B.V., Maastricht, The Netherlands	Multiplex real-time PCR	mtLSU, DHPS mutations	BALF
AusDiagnostics Respiratory panel, pneumonia panel, atypical pneumonia panel	AusDiagnostics Pty Ltd., Mascot, NSW, Australia	Multiplex real-time PCR	Unknown	Swabs, sputum, BALF, tissue, nasopharyngeal aspirate
Bio-Evolution *Pneumocystis*	Bio-Evolution, Brysur-Marne, France	Real-time PCR	mtLSU	BALF
*Pneumocystis* ELITe MGB	ELITechGroup S.p.A, Turin, Italy	Quantitative real-time PCR	mtLSU	Bronchial aspirate, sputum
PneumID	OlmDiagnostics, Newcastle, United Kingdom	Real-time PCR	mtLSU	BALF, washings
Fungiplex^®^ *Pneumocystis* IVD	Bruker Daltonik GmbH, Bremen, Germany	Multiplex real-time PCR	Unknown	BALF, throat swabs
MycoReal^®^ *Pneumocystis*	Ingenetix GmbH, Vienna, Austria	Real-time PCR	mtLSU	BALF
MycoGENIE^®^ *Pneumocystis jirovecii*	Ademtech, Pessac, France	Real-time PCR	mtLSU	Respiratory tract samples
AmpliSens^®^ *Pneumocystis jirovecii*-FRT	Ecoli Dx, s.r.o., Prague, Czechia	Real-time PCR	mtLSU	BALF, sputum, oropharyngeal and tracheal aspirates, lung biopsy, oropharyngeal washes, swabs
RIDA^®^GENE *Pneumocystis jirovecii*	R-Biopharm, Darmstadt, Germany	Multiplex Real-time PCR	mtLSU	BALF
*Pneumocystis jirovecii* (*carinii*) Real-TM	Sacace, Como, Italy	Real-time PCR	mtLSU	Sputum, BALF, tissue, swabs
LightMix Modular *Pneumocystis jiroveci*	Roche Diagnostics, Mannheim, Germany	Real-time PCR	MSG	Unspecified
RealCycler PJIR	Progenie-molecular, Valencia, Spain	Real-time PCR	mtLSU	BALF

BALF: bronchoalveolar lavage fluid; DHPS: dihydropteroate synthetase; mtLSU: mitochondrial large subunit; MSG: major surface glycoprotein.

**Table 3:** Commercially available molecular assays for *Cryptococcus* spp.

Assay	Manufacturer	Method	Target	Samples
*Cryptococcus neoformans* real-TM	Sacace, Como, Italy	Real-time PCR	Unknown	CSF, BALF, sputum, blood, skin lesions aspirate, viscera biopsy and autopsy material
BioFire^®^ FilmArray^®^ Meningitis/Encephalitis (ME) Panel & Blood Culture Identification (BCID) Panel	bioMérieux, Marcy-l’Étoile, France	Integrated extraction and amplification with multiplex PCR and high-resolution melt analysis	Unknown	CSF, blood
Multiplex Tandem PCR (MT-PCR) CSF and Atypical Pneumonia panels	AusDiagnostics Pty Ltd., Mascot, NSW, Australia	Multiplex PCR	Unknown	CSF, swabs, sputum, BALF, tissue, nasopharyngeal aspirate

BALF: bronchoalveolar lavage fluid; CSF: cerebrospinal fluid.

**Table 4:** Commercially available molecular assays for *Candida* and *Candida*-like organisms.

Assay	Manufacturer	Method	Target	Species	Samples
T2Candida^®^	T2 Biosystems, Lexington, MA, USA	Integrated extraction and T2 magnetic resonance	ITS2	*C. albicans*/*C. tropicalis*, *N. glabratus* complex/*P. kudriavzevii*, *C. parapsilosis* complex	Whole blood
AusDiagnostics Sepsis panel	AusDiagnostics Pty Ltd., Mascot, NSW, Australia	Multiplex tandem PCR	ITS1 or ITS2	*C. albicans*, *N. glabratus*, *P. kudriavzevii*, *C. parapsilosis*, *C. tropicalis*	Unknown
CandID^®^ and AurisID^®^	OlmDiagnostics, Newcastle, UK	Multiplex real-time PCR	Target unknown	*C. albicans*, *C. dublinensis*, *N. glabratus*, *P. kudriavzevii*, *C. parapsilosis* and *C. tropicalis* and *C. auris*	Surveillance swabs (axilla/groin, nasopharyngeal), serum, plasma
BioFire^®^ FilmArray^®^ Blood Culture Identification (BCID) Panel	bioMérieux, Marcy-l’Étoile, France	Integrated extraction and amplification with multiplex PCR and high-resolution melt analysis	Target unknown	*C. albicans*, *N. glabratus*, *P. kudriavzevii*, *C. parapsilosis*, *C. tropicalis*	Positive blood culture
FungiPlex^®^ *Candida* and FungiPlex^®^ *Candida auris*	Bruker Daltonik GmbH, Bremen, Germany	Multiplex real-time PCR	Target unknown	*Candida* spp. (*C. albicans*, *C. parapsilosis*, *C. dublinensis*, *C. tropicalis*), *N. glabratus*, *P. kudriavzevii* and *C. auris*	(FungiPlex *Candida*) DNA extract from whole blood, serum, plasma (FungiPlex *Candida auris*) DNA extract from samples
MagicPlex Sepsis Real-Time test	Seegene, Seoul, Republic of Korea	Multiplex real-time PCR	Target unknown	*C. albicans*, *N. glabratus*, *P. kudriavzevii*, *C. parapsilosis* and *C. tropicalis* (and *A. fumigatus*)	Whole blood
MycoReal *Candida* & *A. fumigatus*	Ingenetix, Vienna, Austria	Real-time PCR with melt curve analysis	ITS2	*C. albicans*, *C. dubliniensis*, *N. glabratus*, *P. kudriavzevii*, *C. lusitaniae*, *C. parapsilosis* and *C. tropicalis*, *A. fumigatus*	Whole blood, aspirates, punctates, CSF, BAL, tissue and FFPE
SeptiFast Real-time PCR	Roche Diagnostics, Mannheim, Germany	Multiplex real-time PCR	Target unknown	*C. albicans*, *N. glabratus*, *P. kudriavzevii*, *C. parapsilosis* and *C. tropicalis*	Blood
SepsiTest-UMD	Molzym Molecular Diagnostics, Bremen, Germany	PCR and Sanger sequencing	18*S* rDNA	All fungal species	Whole blood, blood cultures, CSF, BALF, fluids, tissue, swabs, ultrasonic fluids (prostheses)
Sepsis Flow Chip	Master Diagnostica, Granada, Spain	Multiplex PCR and hybridisation with DNA microarray (no specific DNA extraction step required)	ITS2	*C. albicans*/*C. tropicalis*, *N. glabratus* complex/*P. kudriavzevii* and *C. parapsilosis* complex	Blood cultures, rectal exudates, colonies

BALF: bronchoalveolar lavage fluid; CSF: cerebrospinal fluid; FFPE: formalin-fixed paraffin-embedded; ITS: internal transcribed spacer.

**Table 5:** Commercially available molecular assays for Mucorales.

Assay	Manufacturer	Method	Target	Species	Samples
MucorGenius^®^	PathoNostics B.V., Masstricht, The Netherlands	Multiplex Real-Time PCR	28*S* rDNA	*Rhizopus* spp., *Mucor* spp., *Lichtheimia* spp., *Cunninghamella* spp., *Rhizomucor* spp.	BALF, biopsies, paraffin-embedded tissue, serum
MycoGenie^®^ *Aspergillus* spp./Mucorales spp.	Ademtech, Pessac, France	Duplex Real-Time PCR	28*S* rDNA	*Aspergillus* spp., *Rhizomucor pusillus*, *Mucor indicius*, *M. circinelloides*, *M. plombeus*, *Rhizophus arrhizus*, *R. stolonifera*, *Lichtheimia corymbifera*, *L. glauca*, *Cunninghamella bertholletiae* and *Mycotypha* spp.	Serum, biopsies, lower respiratory tract samples
FungiPlex^®^ Mucorales	Bruker Daltonik, GmbH, Bremen, Germany	Real-Time PCR	Target unknown	*Rhizopus* spp., *Lichtheimia* spp., *Cunninghamella* spp., *Rhizomucor* spp., *Mucor* spp., *Actinomucor* spp., *Apophysomyces* spp., *Saksenaea* spp., *Syncephalastrum* spp.	Serum, plasma, whole blood

BALF: bronchoalveolar lavage fluid.

**Table 6:** Main resistance mechanisms and genes involved in *Candida* and *Candida*-like organisms.

Antifungal Class	Molecular Resistance Mechanism	Phenotype
Azoles	*UPC2* or *ERG11* point mutations	Decreased target enzyme (lanosterol 14-ademethylase) affinity for drug
	*ERG3* point mutations	Inactivation of C5 sterol desaturase altering ergosterol synthetic pathway
	*ERG11* upregulation by gene duplication and transcription factor regulation	Increased concentration of target enzyme
	*CDR1/CDR2* and *MDR1* upregulation by point mutations in *TAC1*, *MRR1* and *MRR2* transcription factors	Decreased intracellular drug concentration (efflux pump upregulation)
Echinocandins	*FKS1* and *FKS2* mutations	Decreased glucan synthase

**Table 7:** Main resistance mechanisms and genes involved in *Aspergillus* spp. (adapted from [141,142,143]).

Antifungal Class	Molecular Resistance Mechanism	Phenotype
Azoles	*cyp51A* point mutations	Decreased target enzyme 14α-demethylase affinity for drug
	*cyp51A* tandem repeat in the promoter region with or without accompanying mutations	Increases the protein level of expression and alters the docking of azoles conferring resistance
	Non-*cyp51A*: Overexpression of ATP binding cassette	Decrease in intracellular drug concentrations (efflux pump upregulation)
Echinocandins	*FKS1* mutation dependent-mutations in hotspot regions	BDG synthase enzyme with highly reduced sensitivity to echinocandin drugs
	*FKS1* mutation independent-caspofungin mediated alteration of the glucan synthetase lipid microenvironment and off-target effect on mitochondria leading to increased reactive oxygen species	Alters the enzyme drug-binding affinity

## Abbreviations

BALF	bronchoalveolar lavage fluid
BCID	Blood Culture Identification
BDG	β-D-glucan
CAP	community-acquired pneumonia
CDR	*Candida* Drug Resistance
CLIA	Clinical Laboratory Improvement Amendments
CSF	cerebrospinal fluid
Ct	Cycle threshold
DHFR	dihydrofolate reductase
DHPS	dihydropteroate synthetase
DNA	deoxyribonucleic acid
ECMM	European Confederation of Medical Mycology
EORTC	European Organisation for Research and Treatment of Cancer
FFPE	formalin-fixed paraffin-embedded
FPCRI	Fungal PCR Initiative
HRM	high-resolution melt
HS	Hotspot
IA	Invasive aspergillosis
IFD	invasive fungal disease
ISHAM	International Society of Human and Animal Mycology
ITS	internal transcribed spacer
LAMP	loop mediated isothermal amplification
MDR	Multi-Drug Resistance
MIC	minimum inhibitory concentration
mNGS	Metagenomic next-generation sequencing
MSGERC	Mycology Study Group Education and Research Consortium
mtLSU	mitochondrial large subunit
mtSSU	mitchochondrial small subunit
NAAT	Nucleic acid amplification tests
NGS	next-generation sequencing
NPV	negative predictive value
PCP	*Pneumocystis jirovecii* pneumonia
PCR	polymerase chain reaction
PPV	positive predictive value
qPCR	quantitative polymerase chain reaction
RNA	ribonucleic acid
ROC	receiver operating characteristic
SQLE	squalene epoxidase
TAT	turnaround time
WGS	whole genome sequencing
WNA	whole nucleic acid
